# Legacy of Plant Virology in Croatia—From Virus Identification to Molecular Epidemiology, Evolution, Genomics and Beyond

**DOI:** 10.3390/v13122339

**Published:** 2021-11-23

**Authors:** Dijana Škorić, Silvija Černi, Mirna Ćurković-Perica, Marin Ježić, Mladen Krajačić, Martina Šeruga Musić

**Affiliations:** Department of Biology, Faculty of Science, University of Zagreb, 10000 Zagreb, Croatia; silvija.cerni@biol.pmf.hr (S.Č.); mirna.curkovic-perica@biol.pmf.hr (M.Ć.-P.); marin.jezic@biol.pmf.hr (M.J.); mladen.krajacic@biol.pmf.hr (M.K.); martina.seruga.music@biol.pmf.hr (M.Š.M.)

**Keywords:** biocontrol, chestnut blight, *Cryphonectria parasitica*, chromatography, insect vectors, molecular ecology, phytoplasmas, RNA virus satellites, viroids, water

## Abstract

This paper showcases the development of plant virology in Croatia at the University of Zagreb, Faculty of Science, from its beginning in the 1950s until today, more than 70 years later. The main achievements of the previous and current group members are highlighted according to various research topics and fields. Expectedly, some of those accomplishments remained within the field of plant virology, but others make part of a much-extended research spectrum exploring subviral pathogens, prokaryotic plant pathogens, fungi and their viruses, as well as their interactions within ecosystems. Thus, the legacy of plant virology in Croatia continues to contribute to the state of the art of microbiology far beyond virology. Research problems pertinent for directing the future research endeavors are also proposed in this review.

## 1. Introduction

**Figure 1:**
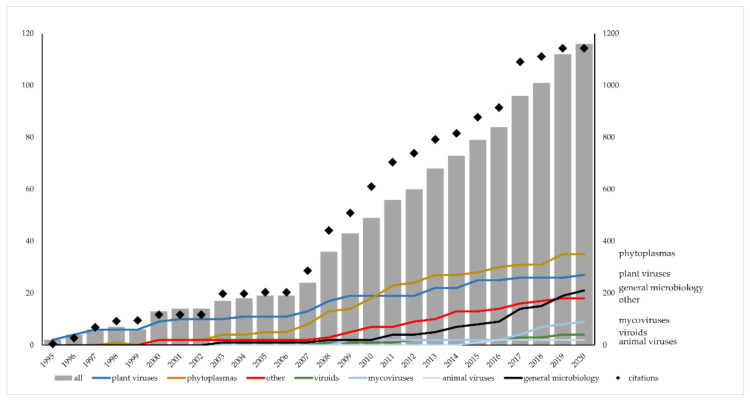
Yearly cumulative numbers of scientific papers and citations accessible in Scopus published since 1995 by the current and previous members of the “plant virus group” (University of Zagreb, Faculty of Science) analyzed by years and by diversified research topics divided into categories. The category “other” contains papers dealing with mixed infections (e.g., viruses and phytoplasmas) or plant physiology and the category “general microbiology” contains those dealing with antibacterials, antibacterial resistance, fungal genetics, or plant-pathogen interactions.

The origin of plant virology in Croatia is traceable back to the year 1943 and the first publication on a plant virus co-authored by Milan Panjan [1], who remained a prominent figure in the field of plant pathology well into the 1970s. Nevertheless, the first modern plant virology laboratory was established in the late 1950s at the Botanical Institute, later to be renamed to Department of Botany, Faculty of Science, University of Zagreb by Professor Davor Miličić. Starting his career as a botanist, he turned to plant virology after finding the paracrystalline inclusions of cactus virus X (CVX) in opuntia cytoplasm [2] and obtaining funding for experimental greenhouses, laboratory equipment, antisera production facility, and research staff. Ana Zlata Štefanac (née Uđbinac) was the first young collaborator who joined him in 1959, followed by Nikola Juretić, Nada Pleše, and Đorđe Mamula. Extensive knowledge of botany, plant anatomy, morphology, and physiology provided a sound foundation for investigations of plant viruses to the research group. Awarded international research fellowships introduced them to the state of the art in the field and ensured their inclusion into the international scientific community. Thus, they formed a hub for plant virus research and education of ex-Yugoslavia. Many colleagues from Sarajevo, Banja Luka, Priština, and Zagreb studied with them as graduate and postgraduate students [3]. This historical review intends to highlight the early achievements of this group and present its legacy by featuring the main research achievements of the latter group members. Their interests diversified further and now cover fields of molecular plant pathology, epidemiology, and evolution, as well as environmental, plant sciences, and biotechnology. Their equally diverse research models encompass not only plant viruses, but also subviral pathogens (viroids, RNA virus satellites), phytoplasmas, fungi, and fungal and animal viruses (Figure 1).

## 2. Early Plant Virus Research

### 2.1. Identifications of Plant Viruses and Their Isolates in Croatia

Until the early 1990s, Miličić et al. led the studies on more than 30 virus species and isolates detected in wild and cultivated plants in Croatia [3] and started establishing the plant virus collection. They were the first to biologically characterize a CVX isolate and its spindle shaped intracellular inclusions [4,5,6,7]. They discovered new strains of radish mosaic virus [8] and tobacco streak virus [9], as well as completely new virus species like Maclura mosaic virus [10] and an ilarvirus from spinach [11]. As serological and biological methods for virus identification were the standard at the time, but viral antisera were not commercially available, they prepared them “in house” for over 40 different viral isolates. Their expertise allowed the improvement of diagnostic techniques as well. This enabled them to succeed in using single radial immunodiffusion tests as both a quantitative assay and to determine the relationships amongst viruses [12]. Interestingly, they were the first to prepare the immune serum against a plant virus in a fish [13].

### 2.2. Cytopathological Changes in Infected Plants

Revealing the plant virus diversity in Croatia and establishing a plant virus collection provided experimental systems for studying virus biology and impact on the infected cells. Their investigations of virus intracellular inclusions observed by light and electron microscopy remain the hallmark of their early work, cited in many older plant virology textbooks and monographs [3] with photographs of their research objects featured in the descriptions of plant viruses (e.g., CVX, https://www.dpvweb.net/dpv/ [accessed on 17 September 2021]). Miličić and collaborators investigated the intracellular inclusion bodies’ submicroscopic structure of Holmes’ ribgrass virus [5], cauliflower mosaic virus [14], radish mosaic virus [15], Narcissus mosaic virus [16], broad bean wilt virus [17], and potyviral pin-wheel inclusions [10]. Probably the most important contribution in this line of investigation was the role of mitochondria in the formation of tobacco rattle virus inclusions, so called X-bodies. Those inclusions, studied in the cells of the experimental plant *Nicotiana clevelandii*, were formed from mitochondria that developed abnormal peripheral membranous sacs and membrane bound vacuoles aggregated with ribosomes and granular material into the X-bodies [18]. These findings paved the way for the observations of mitochondrial abnormalities in animal virus infected cells later.

### 2.3. First Steps towards Virus Ecology

Once established in the laboratory, plant virus study methods were employed for the first investigations of virus presence in the water and soil of various plant habitats in Croatia and Hungary. Horvath et al. [19] and Juretić et al. [20] ultracentrifuged water from the river Danube and Zala in Hungary, respectively, to obtain the inoculum for experimental herbaceous virus hosts from the sediment. Viruses were identified on the basis of host range, symptomatology, intracellular inclusions, morphology of virions and serological reactions. Tobamoviruses, known to have very stable particles, were identified. Besides the tobacco mosaic virus (TMV) previously found in rivers of Yugoslavia, Italy, and the USA [21], a new isolate related to the ribgrass mosaic virus (RiMV) was found in the river Zala [20]. The authors were not surprised by the latter finding, knowing the wide host range of RiMV, the virion stability, and the main routes by which the virus could have been released into the water bodies. Infected plant waste, compost material, and the release of virus particles from infected plant roots are known plant virus transmission routes whose role in plant virus epidemiology tends to be overlooked. However, the importance of these routes seems to be increasing with the modern agricultural practices of sustainable plant production, especially if reclaimed water is used or hydroponics systems are established.

The water–soil interface is equally important in the wider picture of plant virus ecology. Various types of soil, depending on their colloidal complex (charge and adsorption surface area), differentially retain virus particles, thus regulating their release into the water. The absorption of TMV anisometric rodlike virion and turnip yellow mosaic virus (TYMV) with isometric particles was tested in humic and sandy soil samples [22]. The percentage of absorbed viruses was very high—around 95% for TMV and almost 100% for TYMV. Surprisingly, sandy soils absorbed viruses similarly well as the humic ones. That was explained by pulverized soil components with high proportion of colloidal fraction. More importantly, the authors were able to draw conclusions on the virus soil retention dynamics and the virus release into surface water occurring mostly from the upper soil layers.

Pleše et al. [23] investigated viruses in the soil samples containing plant roots from three forest districts near Zagreb, including forest brooks and ditches encompassing different parts of the ecosystems containing plant viruses. Using bait plants and biological indexing on experimental test plants revealed the presence of tobacco necrosis virus (TNV), recently renamed tobacco necrosis virus A (TNV-A, *Tombusviridae*), in 57% of soil samples but not in the nearby water samples. This work disclosed a distinct new strain of TNV, identified by serological and biological methods. On the other hand, the investigated water bodies contained tobamoviruses TMV and tomato mosaic virus (ToMV), but not TNV, as identified by double radial immunodiffusion, bioassays, and electron microscopy. TMV was isolated from water silt, even without a concentration step, indicating its high titer and stability in the environment, known characteristics of tobamoviruses. They hypothesized that the presence of tobamoviruses in the water, but not in the surrounding soil, and vice versa for TNV, was linked to the tobamovirus release into the water from infected, untested herbaceous plants in the same ecological niche and with the easier washing out of TNV from topsoil into the deeper layers. As far as we know, this paper [23] remains the only attempt to investigate plant viruses on the plant–soil–water interface in Croatia.

## 3. Plant Virus Research Models and the Path towards Investigations of Subviral Agents

### 3.1. Multipartite Viruses

Multicomponent (multipartite) species are relatively common among plant and fungal viruses [24]. Divided genome with segments encapsidated in separate virus particles is an intriguing feature of which some aspects are still poorly understood [25]. Their evolutionary advantages and shortcomings remain an issue in virology even nowadays. In the 1970s, pioneering research challenged the assumption of what components had to be co-transmitted to maintain the integrity of a viral species. Nikola Juretić and Robert W. Fulton purified radish mosaic virus and separated it into several components by sucrose density gradient centrifugation [26]. The components were called Ta, T, M, and B, to denote their position from the top to the bottom of the tube with particles in the bottom (B) component being larger than the ones above. Separated components were non-infectious when inoculated alone, except for the B which was slightly infectious. On the other hand, the infectivity was greatly enhanced by mixing components M and B, suggesting that the virus genome consists of RNA segments packaged in separate particles—an attribute of multipartite viruses. The topic is still in the focus of scientific interest as the existence of multicomponent animal viruses was demonstrated only recently [27].

The Croatian isolate of tobacco streak virus (TSV, *Ilarvirus*) from wild shrubs of *Clematis vitalba*, was another investigated multipartite virus [9]. With three classes of quasi-spherical particles, 76S, 87S, and 98S, having RNA species of 0.7, 0.9, and 1.1 × 10^6^, respectively, Da encapsidated by a single 25 kDa polypeptide, and internally transcribed subgenomic RNA, it met the general criteria for a new *Bromoviridae* member [28]. However, it was classified as a distinct TSV strain due to its serological relationships to previously described isolates. As the virus was detected in *C. vitalba* expressing chlorotic spots or yellow netting, as well as in symptomless plants from distant and climatically different regions, the wild *Clematis* host was recognized as a significant reservoir of this seed- and thrips-transmitted virus.

### 3.2. Cucumber Mosaic Virus and Its Satellite RNA

In the following decade, another multicomponent virus seriously endangered, and finally devastated industrial tomato production in the south of Croatia. In fact, cucumber mosaic virus (CMV) was just a helper in a complex virus–subviral agent–tomato network of interactions. An associated satellite RNA (satRNA), a noncoding linear RNA dependent on the helper virus for the replication and spread, was identified as a principal etiological agent responsible for the devastating tomato lethal necrosis disease [29]. Nucleotide (nt) sequencing of cloned cDNAs revealed small differences in variable domains compared to referent necrogenic satRNAs, but almost a perfect fit with a 19-nt region described as “necrogenic consensus” sequence at the 3′-end of other CMV necrogenic satellites [30]. The secondary structure of the satellite was predicted by computer modelling [29] as its structure is the only biological resource possessed by these small non-coding RNA molecules to exert their differential effects on the host. A conserved secondary structure, encompassing biologically active hairpins, was recently identified in ameliorative satRNAs, enabling them to compete with the helper virus for replication resources and, thus, reduce viral replication and symptom expression [31]. Weed species collected from the diseased tomato growing area revealed three main reservoir hosts (*Anagallis arvensis*, *Solanum nigrum*, and *Sonchus* sp.), two of which were perennial plants, likely to maintain the CMV–satRNA complex until its transmission to tomato plants by aphids in the following season [32]. This glimpse into the ecology of CMV+satRNA as the causal agent of tomato lethal necrosis in the Neretva Valley enabled informed decisions of the growers for the management of the disease.

### 3.3. The Application of Monolith Chromatography for the Investigations of Viruses and Subviral Agents

CMV satRNA, amongst the simplest biological entities on the edge of life, will, henceforth, turn out to be the most researched subviral RNA in the Croatian virology. Both the helper and the satellite replication processes generate sufficient quantities of double-stranded RNAs (dsRNAs) to be easily extracted from small amounts of plant tissue utilizing CF-11 cellulose chromatography followed by electrophoresis. This approach is useful in preliminary identification of many plant viruses, and particularly suitable for this harmful virus–satellite combination [33]. The invention of monolithic chromatographic supports has enabled the use of HPLC, a powerful and widely used separation technique, for the assessment of large biomolecules, including virus particles and viral genomes. At the beginning of the new millennium, CMV satRNA was used as our first model to demonstrate the value of the monolith chromatography as a new tool in plant virology [34]. Fast and efficient recognition of pathogens’ presence was accomplished by running total nucleic acid extract from the infected plant tissue through the weak anion exchanger methacrylate monolithic column. Four types of RNA, both virus and satellite single-stranded (ss-) and dsRNAs, were separated from plant DNA. This was a pioneering work in the application of monolith chromatography for the separation of RNA molecules with different conformational features.

As many viruses infecting fungi either possess dsRNA genomes or have dsRNA replicative intermediates, the same chromatographic approach was used to set up an improved purification of mycoviral dsRNA from nucleic acids partially purified by CF-11 cellulose chromatography [34]. The separation of dsRNA directly from total nucleic acids’ extracts enabled fast and simple screening of a virulence attenuating mycovirus in *Cryphonectria parasitica*, the fungal agent of devastating chestnut blight disease.

Unique conformational features were used in analyzing yet another subviral agent—circular, single-stranded, highly-structured potato spindle tuber viroid (PSTVd) RNA. Hydrophobic interaction chemistry turned out to be superior to ion-exchange in chromatographic concentration of this pathogen. By using larger preparative columns, the quick process enabled the concentration of PSTVd RNA by two orders of magnitude with 70% recovery [35]. As described for viruses present in irrigation water, the preconcentration of viroids could contribute to a more sensitive detection of these important subviral phytopathogenic agents.

To prove a presumption that ion-exchange chromatography could separate viruses if they had different electrostatic properties, an artificially mixed sample consisting of rod-shaped TMV, small icosahedral tomato bushy stunt (TBSV), and turnip yellow mosaic virus (TYMV) as models was successfully separated into components [36]. This result implicates a possibility of fast preliminary detection of mixed virus infections by monolith ion-exchange chromatography.

Monolith chromatography has been a prominent part of the Croatian, but also Slovenian, plant virology research with remarkable achievements resulting in several jointly published papers. This offered new possibilities and triggered new aspects of research in virology. A review dedicated to monoliths and their application in virus research rounded off a decade of fruitful collaborative research [37].

## 4. The Research Diversification in the Pregenomic Era

### 4.1. Viroids—The Ultimate Subviral RNA Pathogens

Viroids are independent plant pathogens consisting solely of a short, single-stranded, circular RNA without protein coding capacity. Unlike viruses, that are essentially parasites of the translational machinery of the cell, viroids are parasites of its transcriptional system. They are, therefore, invaluable research objects in RNA structure–function relationships, trafficking, signal transduction, and molecular evolution studies [38,39]. However, they have become the objects of many research initiatives primarily due to the significant damage they cause to various important crops, including citrus. As symptom-based identification procedures are not always suitable for viroids, it is necessary to identify these pathogens through determining their nucleotide sequence and sometimes even secondary structure (circularity and single-strandedness of the molecule). There are eight viroid species known to infect *Citrus* sp., adversely affecting their growth and yield. Amongst them, Citrus exocortis viroid (CEVd) and hop stunt viroid (HSVd) are recognized as the most important ones. Molecular analyses of citrus samples from the Croatian coast and islands confirmed that CEVd, and possibly other citrus viroids, had been introduced into the country with imported plant material. Highly pathogenic variants of CEVd were detected in mixed infections together with other citrus viroids with lower pathogenic potential (e.g., CVd-IIa, CVd-IIb, and Citrus dwarfing viroid—CDVd) in many Croatian citrus accessions [40,41]. This was not surprising because citruses are long-living trees whose serial propagation includes grafting of scion budwood to suitable rootstock material. Thus, viroid mixed infections present substantial challenges in the investigations of citrus viroid pathogenicity.

In order to investigate the complex interactions among CEVd sequence variants and their hosts, an in vitro system for the propagation of CEVd in *Gynura aurantiaca* plants was set up in the laboratory [42]. However, the conclusions drawn from this system are limited, as the host defense responses (e.g., RNA silencing mechanisms) can also be influenced by external factors, such as temperature. This had been previously shown for *G. aurantiaca* and CEVd in a greenhouse setting [43]. As to what determines if the viroid–host combination is going to result in the development of symptoms remains unknown because host–viroid sequence variant combinations may result in infections with severe symptoms or be completely asymptomatic. Some of the latter examples include members of the genus *Coleviroid*, mostly asymptomatically infecting the horticultural crop *Coleus scutellarioides* (syn. *Coleus blumei*, *Plectranthus scutellarioides*). We have reported such asymptomatic infections of *C. scutellarioides* with Coleus blumei viroids (CbVds) in Croatia for the first time in 2019 [44]. Globally present coleus blumei viroid 1 (CbVd-1) was detected, as was, surprisingly, CbVd-3, previously reported only in Germany and China.

### 4.2. Viruses and Their Populations—Molecular Perspective

Citrus tristeza virus (CTV) is the most destructive and economically the most important viral pathogen affecting citriculture. Its presence in Croatia was first reported in 1986 [45]. The CTV-infected germplasm probably had been introduced into nurseries resulting in most of the progeny trees to be infected. After the first studies, based on CTV monitoring by serological tests, methods for molecular detection and characterization of CTV were introduced in our laboratory in the early 2000s. In collaboration with Portuguese colleagues, a large number of isolates from Croatia, and some from Montenegro and Albania, were sequenced and analyzed phylogenetically. Surprisingly, CTV variants from six out of seven described phylogenetic groups were detected in our research, pointing to the East Adriatic as a reservoir region of severe CTV strains in the Mediterranean basin [46]. The phylogenetic analyses confirmed the earlier assumption of the CTV introduction with infected satsuma mandarin seedlings imported from Japan. It also inferred pathogenic potential of the strains later corroborated biologically by graft inoculation on standard citrus indicator plants.

Having in mind the long lifespan of citrus trees, high probability of reinfections due to agronomic practices (e.g., pruning tools contamination and vegetative propagation) and variability of RNA viruses, we started comprehensive analyses of within-host CTV population structure. The approach included separation of virus genomic variants by cloning, followed by their identification and quantification by single-strand conformation polymorphism (SSCP) analysis and sequencing. Heterogeneous population structure was observed in almost all samples. Most importantly, we established that severe CTV symptoms may be linked to virus variants with low abundance in intra-host populations [47], as previously demonstrated in some human viruses. As minor virus variants and their pathogenic potential might be overlooked by the routine analyses, a mathematical model determining the minimal sample size, applicable in the study of various viruses, was proposed [48]. Major aspects of our citrus research, including the aforementioned CTV diagnostic approaches, viroid pathogens, in vitro-based sanitation methods, and biological characterization of monophyletic CTV isolates, have recently been covered in a comprehensive review [49]. Valuable experience gained from CTV research was used in the application of well-established methodology for studies of other plant and animal pathogens, like plum pox virus [50], grapevine leafroll-associated virus 3 [51], and hepatitis E virus, an emerging, but still insufficiently researched zoonotic virus [52].

### 4.3. Trilateral Interactions of a Plant, a Phytopathogenic Fungus and a Mycovirus

**Figure 2:**
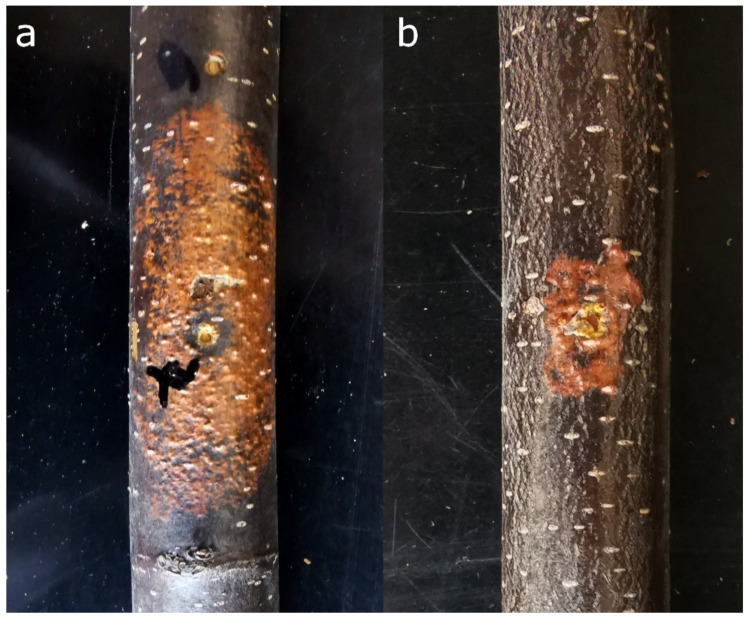
Lesions induced by inoculations of a virulent strain of *Cryphonectria parasitica* (**a**) and its isogenic, but hypovirulent, strain (**b**) infected with Cryphonectria hypovirus 1 (CHV1) in stem sections of European chestnut (*Castanea sativa* Mill.).

Experience in the research of plant viruses enabled new scientific directions encompassing phytopathogenic fungi and mycoviruses, some of which can attenuate virulence of their fungal hosts, thus making them useful as potential biocontrol agents. Today, Cryphonectria hypovirus 1 (CHV1) is known as one of the most successful examples of a biological agent successfully controlling chestnut blight, a plant disease caused by the invasive fungus *C. parasitica*. The infection of *C. parasitica* with this +ssRNA unencapsidated virus attenuates fungal growth within the chestnut bark (Figure 2) and induces healing of the diseased chestnuts [53]. Studies on genetic diversity of the virus defined several subtypes of CHV1 in Europe: the Italian (I) in southern and eastern; two French, F1 and F2, in western; and German/Spanish, D/E, in western and central Europe; as well as Georgian subtype, G, in the Caucasus [54]. Research on CHV1 genomic characteristics performed by our team revealed that most subtypes, except the Italian, are closely related and form a distinguished Western clade, while the viruses of the Italian subtype formed their own clade. Furthermore, Georgian subtype was shown to be a result of a recombination event that probably occurred in the viruses’ native range in eastern Asia before it was introduced to the Caucasus region. Quite interestingly, significant purifying (negative) selection has played an important role in the evolution of the different CHV1 subtypes as well [55].

The severity of the observed effects of CHV1 on its host fungus and chestnut blight is presumed to be dependent on several factors, including the viral subtype, fungal isolate, and the host tree genotype [56,57]. Various CHV1 subtypes, particular virus strains, and fungal isolates were used in different combinations to determine the effect of each of these factors. These combinations were assessed by inoculations on chestnut stems by Krstin et al. [58], revealing that some CHV1 isolates belonging to different subtypes can affect *C. parasitica* similarly, contrary to the established paradigm. Furthermore, the infection with CHV1 affected the general methylation pattern of *C. parasitica* genome [59]. Usually it increased the number and diversity of methylation-sensitive amplification polymorphism (MSAP) markers compared to isogenic uninfected controls [59]. The infection with different CHV1 strains mostly decreased in vitro growth rate of infected fungal isolates and increased activity of fungal stress enzymes in most of the studied fungus/virus combinations, indicating increased oxidative stress following CHV1 infection [60]. Furthermore, we have shown that infection with CHV1 affected the activity of *C. parasitica* laccases, enzymes that degrade lignin and take part in other cellular processes, like morphogenesis, fungal/plant interactions, stress defense, etc. Some CHV1 strains increased the activity of *C. parasitica* laccases of certain fungal isolates upon infection, which is contrary to previous assumptions, and that effect is strongly influenced by culture conditions and fungal/viral genotypes [61].

Field studies in Slovenia and Croatia reveal that chestnut stands began to recover as the consequence of biological control of the chestnut blight fungus induced by naturally occurring CHV1 [62,63]. The virus was present in all sampled *C. parasitica* populations, although with quite variable prevalence. All CHV1 isolates in Croatia and Slovenia belonged to Italian subtype of the virus [62,63,64], presumed to have spread with its host from the neighboring regions. The genetic diversity of *C. parasitica*, measured as the number and proportion of vegetative compatibility types of the fungus within a population, was high as well [63,64,65]. This is considered an obstacle for the spread of the virus, since mycelia belonging to different vegetative compatibility groups cannot form stable anastomoses between their hyphae, thus hindering the successful horizontal spread of the CHV1 within a population. Therefore, dynamics of *C. parasitica* and CHV1 populations have been carefully monitored over the years, uncovering new vegetative compatibility types in Croatia and variability in CHV1 prevalence [66]. Therefore, the persistence of already established natural biocontrol was studied, too [65]. Our long-term studies which included genetically diverse populations of *C. parasitica* from Croatia and Switzerland and mostly clonal ones from North Macedonia, revealed that natural biological control is fairly robust and CHV1 can stay prevalent in a population despite an increase in genetic diversity of its host fungus [67].

Our team continues to study CHV1 especially focusing on its genetic variability and bottleneck effect of viral transmission and its consequences on the host. Early experiments have established a new and improved method for studying a large number of viral variants across their host populations and indicated higher intra-host CHV1 diversity than previously assumed (unpublished results).

### 4.4. The Other End of the Spectrum—Phytoplasmas, Prokaryotes Unusual Enough to Be Mistaken for Viruses

The oldest documentation of phytoplasma diseases dates back to some 1000 years ago when unusual peonies with delicate green petals were admired and presented at the Chinese imperial court [68]. Nonetheless, it took almost another millennium to associate attractive greening of flowers with phytoplasma infections. Prior to discovery of these pleiomorphic bacteria resembling mycoplasmas in phloem sieve elements of dwarfed and yellowing plants in 1967 [69] using electron microscopy, many plant diseases with similar symptoms were considered to be caused by unknown insect-transmissible viruses. The discovery of mycoplasma-like prokaryotes made a breakthrough and initiated the etiology re-examination of plant diseases previously assumed to be caused by viruses [70]. Croatian plant virologists and pathologists at that time followed that trend. They reported data on symptoms of tomatoes, peppers, and potatoes, considered to be caused by the “stolbur virus” [71], followed by the evidence of mycoplasma-like organisms presence, revealed by electron microscopy [72]. Diseases in fruit trees, such as pear decline (PD) and apple proliferation (AP), were also reported [73]. The phytoplasma etiology was confirmed by electron microscopy and oxytetracycline treatment [74].

Typical symptoms of grapevine yellows (GY) in Croatia were observed in the mid-1990s, and the first molecular evidence of the phytoplasmas presence in grapevine was given by Šarić et al. [75]. Since then, systematic monitoring and molecular investigations of GY pathosystems have been implemented. Phytoplasma belonging to the ribosomal subgroup 16SrXII-A (*stolbur*; *bois noir*), classified as ‘*Candidatus* Phytoplasma solani’, was the first etiological agent associated with GY [76,77]. It remains widespread and present in most of the Croatian grapevine growing regions.

Molecular epidemiology of *bois noir* (BN) phytoplasma has been extensively studied over the years, including the identification of the main insect vector *Hyalesthes obsoletus* Signoret 1865, alternative vectors from the genus *Cixius*, as well as plant reservoir species belonging to the grapevine pathosystem. Detailed molecular characterization of BN isolates from all winegrowing regions using multilocus sequence typing (MLST) approach has shown epidemiologically significant diversity of phytoplasma strains in Croatia and their diverse geographical distribution. At least 19 different comprehensive genotypes were recorded, with one being prevalent and widespread in the central north-western part of Croatia. Furthermore, identification of numerous different genotypes unveiled tremendous complexity of BN pathosystems and suggested the presence of several independent epidemiological cycles involving this phytoplasma in Croatia [78,79,80,81,82].

Another phytoplasma associated with GY diseases, a devastating quarantine pathogen belonging to 16SrV ribosomal subgroup, *flavescence dorée* (FD) phytoplasma, transmitted by insect vector *Scaphoideus titanus* Ball, was detected in a grapevine for the first time in 2009 [83]. It immediately raised alert and strict phytosanitary measures were undertaken. Despite the efforts, the disease spread rapidly to most of the winegrowing regions. Finding heavily affected areas in Istrian peninsula and north-western Croatia prompted continuous monitoring of the disease and identification of FD phytoplasma strains. Over the years, more than 800 grapevine samples, together with potential reservoir plants and almost 400 samples of insects, including *S. titanus* and other potential vectors, were collected and analyzed by MLST approach. In total, 15 comprehensive FD genotypes were identified, with the presence of all three mapFD types among the strains. Furthermore, for the first time, FD phytoplasma was detected in an invasive tree species *Ailanthus altissima* Mill. and in a new potential insect vector *Phlogotettix cyclops*, as well as an FD-related phytoplasma in alder [84]. Finding distinct FD clusters and different distribution of genotypes based on MLST suggests separate routes of introduction and spread of the disease and indicates complex epidemiological cycles and ecology of this pathogen [84]. Sporadically, without much epidemiological relevance, phytoplasmas from the 16SrI-B subgroup (aster yellows; AY) attributed to the ‘*Ca.* P. asteris’ species were found in ‘Plavac mali’ vines from Pelješac peninsula and ‘Moslavac’ from Voloder.

Surveys on the presence and diversity of phytoplasmas infecting fruit trees and their vectors in Croatia were also initiated in the early 2000s. Even the first results revealed much higher phytoplasma diversity in fruit trees and vectors than anticipated, with phytoplasmas belonging to ‘*Ca.* P. pyri’ (16SrX-C subgroup) and ‘*Ca.* P. prunorum’ (16SrX-B subgroup) being the most widespread with the highest incidence in pears and stone fruits, respectively. ‘*Ca.* P. asteris’ (16SrI-B) was found sporadically in both fruit trees and vectors, while ‘*Ca*. P. solani’ (stolbur; 16SrXII-A) was frequently found in pear and *Cacopsyla pyri*, the main vector of pear phytoplasma [85]. Although the occurrence of AP, based on symptomatology and electron microscopy of the leaf mid vein sieve tubes of infected trees, was first reported by Šarić and Cvjetković in 1985 [74], first molecular detection and identification of ‘*Ca*. P. mali’ as the etiological agent of AP in Croatia was conducted in 2011 [86]. Psyllid species *Cacopsylla picta* was also confirmed as the only insect vector harboring AP phytoplasma [87]. Molecular epidemiology of AP was further studied by newly established MLST approach encompassing apple and *C. picta* samples from continental and Adriatic regions of Croatia. This study revealed great variability among AP strains with the identification of new and unique genotypes together with previously known ones for all of the analyzed gene regions [88]. Twenty comprehensive allele profiles were identified and assigned to sequence types (ST) 1–20 in order to establish a new MLST scheme that could be widely applied in the future studies. Moreover, ST1 was found to be prevalent in apple samples while ST2 was more frequently found in insects [87].

Phytoplasmas have also been associated with infections of forest and ornamental trees. *Populus nigra* L. ‘Italica’ trees found in the urban area of Zagreb showing symptoms of leaf yellowing, overall sparse foliage, stunting, and decline were infected with phytoplasma from the aster yellows group (16SrI; ‘*Ca.* P. asteris’). Phylogenetic analyses of 16S rDNA as well as other conserved regions revealed that this is a molecularly distinguishable phytoplasma belonging to a newly described subgroup 16SrI-P and rp-O [89]. ‘*Ca.* P. pini’ was detected in *Pinus halepensis* and *P. mugo* trees showing yellowing, proliferations, and shedding of the needles, or yellowing and dieback of shoots and branches, respectively. It had 100% sequence identity with 16S rDNA sequences of ‘*Ca*. P. pini’ previously reported in Spain, Poland, and the Czech Republic [90]. More detailed molecular characterization has been performed on ‘*Ca*. P. ulmi’ isolates from infected *Ulmus laevis* and *U. minor* trees. This phytoplasma is the causal agent of elm yellows and was found to be widely distributed across elm populations in Croatia. Croatian isolates are closely related to strains of ‘*Ca.* P. ulmi’ from southeastern Europe with the presence of new genotypes in analyzed genes [91]. Recently, for the first time in Croatia a phytoplasma from 16SrIX-C group was found in symptomatic *Chicorium endivia* L. and *Zea mays* L. plants [92], strongly suggesting that the discovery of new phytoplasma species, plant hosts and insect vectors is to be expected in future.

## 5. Current Trends in the -Omics Era

### 5.1. From Comparative and Functional Genomics of Phytoplasmas towards Deciphering Pathogenicity Mechanisms and Evolution

The advent of high throughput sequencing (HTS) and a continuous improvement of various related technologies have revolutionized many aspects of plant pathogen research including discovery and routine detection of viruses and viroids [93] or rapid detection of phytoplasmas [94]. Furthermore, the access to full or draft genomes of plant pathogens enabled new insights into their evolution, classification, and, particularly, in putative virulence factors crucial for deciphering mechanisms of pathogenicity.

In our case, phytoplasma research has paved the entrance path to the metagenomic era with virus investigations beginning somewhat later within the scope of international collaborations (https://www.cost.eu/actions/FA1407/ [accessed on 16 November 2021]). As phytoplasmas cannot be successfully grown in pure culture, sequencing and assembly of full genomes are still challenging [95]. Nevertheless, ten phytoplasma genomes have been completely sequenced and assembled so far (https://www.ncbi.nlm.nih.gov/genome/browse#!/overview/candidatus%20phytoplasma [accessed on 29 October 2021]), while a substantial number of genome drafts is also available. A Croatian team contributed to phytoplasma genomic research by obtaining the most complete draft of ‘*Ca*. P. solani’ strain of 821,322 bp, corresponding to more than 80% of the estimated genome size [96]. Detailed comparative and functional analyses of gene content confirmed the presence of some common features of phytoplasma genomes, but also revealed the existence of specific ones that made this genome a highly dynamic and prone to adopting foreign sequences. Putative secreted/effector proteins were predicted setting a foundation for future studies in order to elucidate their specific function and interaction with plant or insect hosts. Moreover, new findings of this study greatly contributed in providing a base for genome-scale genotypic analysis inciting further analyses, particularly within ‘*Ca*. P. asteris’ species [97], as well as evolutionary studies of phytoplasma replisome genes [98].

### 5.2. Viromes of the Plants and Beyond

**Figure 3:**
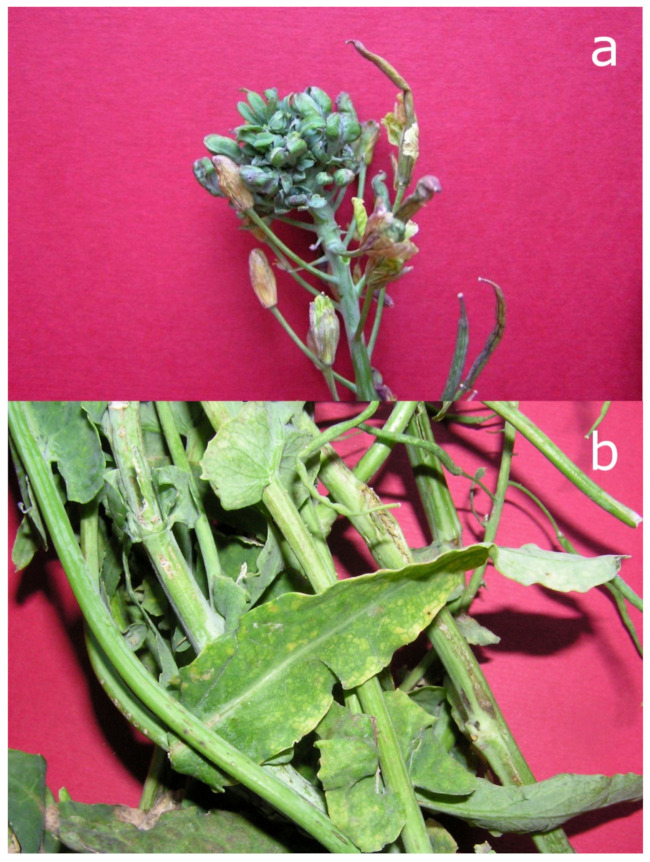
One of the oilseed rape plants (*Brassica napus* ssp. *oleifera* (DC.) Metzg.) showing phyllody of the flowers, greening, stunting, and seed pod deformations associated with the infection of (**a**) aster yellows phytoplasma (‘*Candidatus* Phytoplasma asteris’). Turnip mosaic virus (TuMV) was present in the same plants and lower parts of them (**b**) display leaf chlorosis, necrosis, and stem streaking characteristic of the viral infection.

Our first attempts to investigate the complete repertoire of diverse pathogens associated with a certain plant disease, aside from the chestnut–*C. parasitica* fungus–CHV-1 pathosystem, encompass pre-metagenomic examinations of oilseed rapes (*Brassica napus* L. ssp. *oleifera* (DC.) Metzg) [99]. In severely diseased plants with symptoms reminiscent of phytoplasmoses (virescence, phyllody, big buds, and shortened internodes), not only a member of the ‘*Ca*. P. asteris’ species was identified and characterized but also an important and globally distributed virus of cruciferous plants. This was the turnip mosaic virus (TuMV) typically causing leaf chlorosis, necrosis and stem streaking (Figure 3). It is probably the most widespread plant virus with a very wide host range in subtropical and temperate climatic zones [100] and we proposed it played a role in the severity and type of symptoms displayed in these oilseed rapes [99].

We have only started to harness the power of HTS to investigate the plant–pathogen interaction networks including viruses, their different combinations, phytoplasmas and viroids, or even other types of hosts and their viromes. The participation in the COST Action DIVAS (Application of next generation sequencing for the study and diagnosis of plant viral diseases in agriculture, https://www.cost.eu/actions/FA1407/ [accessed on 16 November 2021]) facilitated this advancement [101]. The RNA sequencing-based approach we apply starts to uncover multiple viruses and viroids coinfecting citrus samples displaying deteriorating symptoms, but also enabled the discovery of novel viruses in the invasive signal crayfish [102]. By using this approach, we have also revealed an interesting syndrome in *C. scutellarioides* with predominating fasciation and witches’ broom symptoms harboring a virus–viroid–phytoplasma combination [103]. This and other complex pathosystems are currently under investigation including vegetable plants, fruit trees, and ornamentals.

### 5.3. Back to the Roots—The Untapped Potential of Plant Virus Collection for Phylogeography and Evolution

Whilst we have only remnants of the couple of hundreds of vials of the plant virus antisera collection at the Department of Biology, the current number of virus isolates in our plant virus collection is 1229. The first isolates date back to the 1970s. The first accession is TuMV from *Brassica rapa* var *rapifera* “purified by a single lesion method” from experimental tobacco, stored on 12 March 1970. The number of isolates changes yearly depending on the introduction of new ones into the collection, using up some of the isolates for research or discarding the old ones due to the loss of infectivity. The isolates are kept in glass tubes as lyophilized tissue on dry silica gel at 4 °C in the dark. Most of them come from infected leaf tissue of herbaceous experimental hosts like *Nicotiana benthamiana*, *N. megalosiphon*, *N. glutinosa*, *Chenopodium quinoa*, *Ch. amaranticolor*, or others, depending on the host range and the most suitable propagation host(s) for each virus. Unfortunately, this collection is not curated and there are many isolates that still lack proper characterization and species, or strain, annotation.

Regardless of the hardships in maintenance, contemporary and old TuMV isolates from the collection were used successfully in our research [99,100]. Many old TuMV isolates from the Zagreb plant virus collection have over 50 years now. A subset of them was tested for their infectivity about a decade ago. The oldest from the subset found to retain its infectivity was from the year 1974. Its full genome sequence shows it is a unique recombinant strain between phylogenetic TuMV world-B3 subgroup and Asian-BR group. Therefore, this and a few other Croatian isolates provided some pieces of the puzzle reconstructing TuMV phylogeography and evolutionary history supporting its spread along the Silk Road [100]. The fact that TuMV isolate from 1974 retained its infectivity for almost 47 years in less than perfect conditions is amazing in itself and supports the notion of notorious TuMV longevity.

Old virus and viroid isolates from institutional collections and herbaria have been successfully used in contemporary research and the introduction of modern research tools, including HTS, hugely drives these changes [104]. Therefore, old isolates have proved invaluable in discovering new virus species, resolving taxonomy questions, and giving new insights into virus diversity, biology, evolution, and ecology [105,106]. Apart from the research mentioned above and using the collection isolates for teaching, the potential of our virus collection is almost untapped. As suggested recently [107], counter intuitive as it may seem, the efforts to conserve plant pathogens hold much value for the future of plant health. They could be used in biotechnology, sustainable plant production, and prove to be important for food and feed security. Currently, we have been trying to “revive” some of the old isolates from the collection [9], potentially representing new species or having unresolved taxonomical status. As the conservation of the collection is a prerequisite for its further usage, our future efforts will be devoted to this goal. The archived virus isolates, as one of the tangible aspects of our legacy, should be validated further, preferably by employing HTS, and will be an open field for our future research.

## Data Availability

All data obtained in this research is available upon request.
